# Innate Immune Memory: The Latest Frontier of Adjuvanticity

**DOI:** 10.1155/2015/478408

**Published:** 2015-08-25

**Authors:** Elfi Töpfer, Diana Boraschi, Paola Italiani

**Affiliations:** Institute of Protein Biochemistry, National Research Council, 80131 Naples, Italy

## Abstract

Recent findings in the field of immune memory have demonstrated that B and T cell mediated immunity following infections are enhanced by the so-called *trained immunity*. This effect has been most extensively investigated for the tuberculosis vaccine strain Bacillus Calmette-Guérin (BCG). Epidemiological studies suggest that this vaccine is associated with a substantial reduction in overall child mortality that cannot be solely explained by prevention of the target disease but that it seems to rely on inducing resistance to other infections. Upon infection or vaccination, monocytes/macrophages can be functionally reprogrammed so as to display an enhanced defensive response against unrelated infections. Epigenetic modifications seem to play a key role in the induction of this “innate memory.” These findings are revolutionising our knowledge of the immune system, introducing the concept of memory also for mammalian innate immunity. Thus, vaccines are likely to nonspecifically affect the overall immunological status of individuals in a clinically relevant manner. As a consequence, future vaccine strategies ought to take into account the contribution of innate memory through appropriate design of formulations and administration scheduling.

## 1. Introduction

Vaccination is the most effective medical intervention introduced within the last 300 years. Its effectiveness results in a reduction of mortality and an increase of life expectancy by the prevention of contagious diseases. A recent report shows that vaccines prevented more than 100 million cases of disease over the last century in the United States alone [1], and every year immunisation programs save 2.5 millions of lives worldwide [2]. Vaccination started as an empirical approach until the emergence of more sophisticated technologies (from recombinant DNA to reverse and structural vaccinology) that allowed more specific and safer formulation of vaccines [3]. One of the challenges of vaccinology has been and still is the development of vaccines that improve and support immature, failing, and compromised immune system in immunologically frail population groups such as newborns, elderly, and chronically ill patients, respectively. Adjuvants have been crucial for vaccine success. Adjuvants are immunostimulatory molecules, such as aluminium phosphate or hydroxide salts (known as Alum), Toll-like receptors agonists (TLRa), such as monophosphoryl lipid A (MPLA) and CpG oligonucleotides, emulsions (e.g., oil-in-water emulsions such as MF59 and AS03), combinations of TLRa with Alum (e.g., AS04), and liposomes/nanoparticles [4, 5]. The name adjuvant (from Latin* adiuvans* = the one who helps) underlines the ability of these agents to help the development of an adaptive immune response against a vaccine antigen by inducing a mild innate inflammatory response [6]. Over 50% of vaccines either licensed or in clinical trials are formulated with adjuvants. The role of adjuvants in inducing effective immunisation has recently been discussed in several extensive reviews [7–9].

In the last years, important discoveries changed the way of looking at the innate immune system. Features as specificity and memory, the main traits of the adaptive immune system, are now also considered to some extent for innate immunity.

The discovery of Patter Recognition Receptors (PRRs) has introduced the concept of specificity in innate recognition, although not in the highly specific fashion characterising adaptive immune recognition. The existence of different classes of innate receptors (such as TLR, C-type lectin receptors (CLRs), nucleotide-binding oligomerisation domain-like receptors (NLRs), and retinoic acid-inducible gene I (RIG-I) helicases) allows innate immune cells to identify different pathogenic microorganisms based on the recognition of pathogen-associated molecular patterns (PAMPs). The discovery of TLR and elucidation of their functions has led to the selection of a new class of adjuvants, that is, the TLR agonists [10–12].

Revisited old knowledge on the repeated stimulation of the innate immune responses has reintroduced the old concept of innate immune memory [13, 14], redubbed “trained immunity,” as proposed by Netea et al. [15].

Evidence in both plants and invertebrates (that do not possess adaptive immunity and classical memory) indicates that phagocytes can respond much better to a challenge if they have been prestimulated with the same or with another agent [16]. Thus, innate immunity can have a memory, although different from acquired immune memory. Recently, “memory” of innate immune cells has been observed in vertebrates [17].  Table 1 summarises the main differences between innate and adaptive memory.

The concept of innate memory might help to develop new strategies of adjuvanticity in the near future.

Generally, vaccines have antigen-specific protective effects, but they can also improve the resistance to other infectious diseases. This phenomenon of nonspecific memory induction may go both ways, as we will better describe later; that is, it can also result in decreased reactivity to an unrelated subsequent challenge. Accordingly, a vaccine is not only a preventive strategy that improves the immune response against a specific infection, but a “biological preparation that alters the resistance towards unrelated pathogens” [18]. Interestingly, recent data reveal that trained immunity/innate memory accounts for nonspecific effects of vaccines along with the well-known role of T and B cell mediated adaptive immunity [18, 19]. Actually, innate immune memory is not a recent discovery in vaccinology, although only recently it has gained a wide interest in the context of the mechanisms underlying the activation of protective immunity.

This review summarises the current knowledge and hypotheses on innate immune memory and its role on vaccine efficiency, focusing on mononuclear phagocytes as the main innate immune cells involved and on the role of innate immune memory on nonspecific immunity. We will also highlight which questions are still unanswered.  Table 2 defines some properties of the immune system, which are mentioned throughout the review.

## 2. Role of Innate Immune Memory in Nonspecific Vaccination Effects

Some vaccines have been associated with a high decrease in mortality that not only is accounted for by their specific effects against a certain pathogen, but also depends on the induction of a nonspecific protection against unrelated infections and pathogens [18]. This nonspecific effect, most likely mediated by both T cell cross-reactivity and innate memory induction, has been extensively investigated for the tuberculosis vaccine strain Bacillus Calmette-Guérin (BCG) [20–23]. The BCG vaccine has been associated with an overall reduction in mortality [18, 24]. In developed countries, in which mortality rates are low, BCG vaccination is related to decreased morbidity outcomes, such as sepsis-related hospitalisation or melanoma risk [23, 25]. Positive nonspecific effects on mortality and morbidity in high and low income countries have been reported also for other live vaccines, for example, against measles [26–28] and smallpox [29]. Conversely, negative effects were observed for inactivated vaccines such as the diphtheria-tetanus-pertussis (DTP) vaccine [30]. In a nutshell, live vaccines are accompanied by positive nonspecific effects, while inactivated vaccines may in some circumstances induce negative outcomes. Time and sequence of vaccine administration and sex of the vaccinees apparently influence the possibility of negative nonspecific effects, at least in less-developed countries [31]. These observations underlie the need of designing appropriate immunisation schedules, aiming at using vaccination to its greatest benefit by optimising efficacy and reducing the possibility of nonspecific deleterious effects [32].

As already mentioned, the favourable nonspecific effects of vaccines are presumably mediated by both adaptive and innate immunity. A study on SCID mice (which are devoid of T and B cells) clearly shows BCG induced nonspecific protection against an unrelated pathogen, thereby underlining the crucial role of innate immune mechanisms in the BCG induced protection [17]. Moreover, the same study reports BCG-dependent trained memory induction in human circulating monocytes, assessed as increased inflammatory cytokine release upon stimulation with unrelated pathogens, and shows that this effect is associated with epigenetic modifications. This trained memory state persisted for at least 3 months [17]. Likewise, NK cells from BCG-vaccinated individuals show an increased inflammatory cytokine release upon* ex vivo* stimulation up to 3 months after immunisation [33]. Interestingly, a study on nonspecific effects of BCG vaccination on subsequent endotoxemia did not show any immunomodulatory capacity of the vaccine [34]. It should be noted that the BCG vaccine used in the study was an inactivated *γ*-irradiated BCG vaccine. Considering that live BCG is detectable for up to 4 weeks at the challenge site [35], it is conceivable that the different immunomodulatory properties of the two vaccines depend on the bacterial persistence (prolonged for the live bacteria, reduced for the inactivated vaccine). In line with this, the capacity of live BCG to induce trained memory in mononuclear phagocytes might vary depending on variations during the production of the vaccine, as a very recent study found elevated memory induction in monocytes from slow growth rate BCG compared to BCG batches with normal growth rates [36].

## 3. Innate Immune Memory: Cells and Mechanisms Involved

A very interesting notion is that the innate memory is at least in part nonspecific, which implies that an improved defensive response can be obtained by prechallenging the host with (almost) any kind of agents. This concept breaks the current dogma that innate immunity is a stable and nonvariable type of response, always the same at every challenge, as opposed to acquired immunity that “learns” after the first encounter and generates more rapid and more efficient responses upon subsequent challenges due to the presence of memory cells.

The mechanisms underlying trained innate immunity have not been fully elucidated. Among the innate immune cells, the most active innate memory cells are monocytes/macrophages and NK cells. Both are cells with low turnover rates and thus more easily trainable compared, for instance, to terminally differentiated and short-lived neutrophils. In mice, memory NK cells mediate protection against viral infections in a T and B cell-independent manner, and memory properties apparently depend on a differential expression of the virus-specific LY49H receptor [37]. Moreover, hepatic CXCR6^+^ NK cells of T and B cell-deficient mice develop nonspecific memory upon vaccination with structurally diverse antigens [38]. In humans, NK cell memory has been observed after cytomegalovirus infection [39]. Recently, it also has been demonstrated that cytokine combinations including IL-12, IL-15, and IL-18 can induce memory-like properties in human [40] and murine [41] NK cells.

It is very interesting that monocytes/macrophages are able to develop different kinds of memory depending on the type of priming. Thus, monocytes/macrophages can develop a memory that leads them to be less reactive to some challenges (tolerance, to avoid extensive tissue damage) or to an enhanced response (training, to improve tissue surveillance, e.g., against tumours). These different ways depend on the nature of the first challenge. Both mild and severe stimulations with LPS trigger a strong reaction but, upon a second challenge, macrophages react much less because they aim at avoiding an excessive reaction to a minor challenge and the consequent risk of unwanted tissue damage [42]. On the other hand, challenge with fungal components and ultralow LPS stimulation (implying a long-term slow infection with tissue debilitation) induces an innate memory that results in enhanced reactivity to subsequent stimuli, necessary for the adequate defense of a weakened tissue [43, 44]. One mechanism that has been identified as possibly underlying this trained memory is the epigenetic reprogramming of monocytes during their differentiation into macrophages, or during LPS tolerance and trained memory effects [44–46]. Some epigenetic markers have been identified that are associated with the acquisition of a trained or a tolerant phenotype, such as trimethylation of the histone 3 (H3) lysine at position 4 (H3K4me3) and acetylation of the H3 lysine at position 27 (H3K27ac) [17, 44]. Epigenetic reprogramming may be induced after infection and vaccination, and innate memory leading to enhanced reactivity can explain at least in part the BCG-induced nonspecific protective properties. H3K4me3 is associated with the trained memory-inducing effect of BGC vaccination in monocytes, an effect that involves the intracellular PRR NOD2 [17]. Moreover, the trained memory induced by BGC on human monocytes persists for at least 3 months after vaccination, with some of the protective effects lasting up to 1 year [33].

The question that arises from these observations is how can monocytes, which possess a relatively short half-life in circulation, be responsible for this long-term protection? A possible explanation is that a reservoir of epigenetically modified monocytes (memory monocytes) persists in the body, possibly located in the spleen, as hypothesized for NK cells. Alternatively, monocyte precursors could be “trained” directly in the bone marrow by the local microenvironment. The latter hypothesis is supported by a recent work that demonstrates how TLR2 stimulation of myeloid progenitor cells can influence the functional phenotype of the macrophages that develop from them [47]. Thus, maintenance of epigenetic modifications can occur during myelopoiesis in the bone marrow, thereby having the potential to influence myeloid cell functions for longer periods.

Other interesting aspects of trained memory are changes in metabolic processes, as already observed in macrophage polarisation [48]. Recent evidence underlines the importance of metabolism in shaping the functional phenotype of macrophages in response to distinct polarising stimuli in the tissue microenvironment, under normal conditions, and in pathological settings [48–51]. Whereas different metabolic pathways are apparently involved, the glucose metabolism seems to play a major role in both polarised and memory macrophages. In response to inflammatory stimuli, macrophages display a metabolic shift towards an aerobic glycolytic pathway (with the transformation of pyruvate to lactate and the rapid energy production, similarly to anaerobic glycolysis), as opposed to the classical aerobic glycolysis (oxidative phosphorylation of pyruvate in mitochondria, with lower rates of energy production) that occurs in alternatively activated macrophages. Likewise, induction of monocyte trained memory by *β*-glucan requires a metabolic shift towards the high energy-producing type of aerobic glycolysis, which is referred to as the “Warburg effect” [52]. The switch to the Warburg type of glycolysis seems to depend on the activation of mTOR through the Dectin 1-AKT-HIF1*α*-dependent pathway [52].

In addition to epigenetic and metabolic reprogramming, other putative mechanisms involved in establishing monocyte memory include the involvement of different monocyte subpopulations (e.g., CD14^dim^CD16^+^, CD14^+^CD16^−^), a topic that has not yet been fully investigated [15]; an increased expression of PRRs on the cell membrane following BCG vaccination [17, 53]; and the role of soluble mediators, such as inflammatory cytokines. The latter mechanism is supported by the fact that peripheral inflammation can modulate immune response in the central nervous system despite the inability of microbial components (such as LPS) to pass the blood-brain barrier [54]. Moreover, plants possess the ability to develop SAR, “systemic acquired resistance” [16, 55], mediated by soluble factors. It is tempting to speculate that similar principles apply also to the innate memory of mammals.

**Figure 1:**
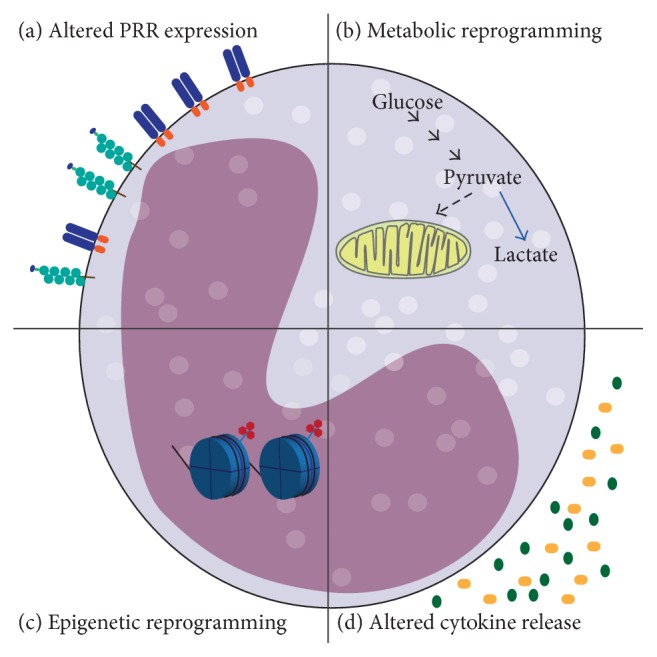
Main mechanisms involved in trained immune memory. In the picture the main mechanisms believed to underlie innate memory are shown. (a)* Altered PRR expression*. Phenotypic changes of innate immune cells with memory properties involve increased expression of PRRs on the cell surface and improved pathogen recognition. (b)* Metabolic reprogramming*. Innate immune memory requires a metabolic shift, which involves Warburg metabolism. The metabolism of glucose is shifted toward increased glycolysis with production of lactate and decreased oxidative phosphorylation. (c)* Epigenetic reprogramming*. Trimethylation of H3 at lysine 4 (H3K4me3) is a marker of promoter activation for proinflammatory genes specifically induced by *β*-glucan-dependent memory. (d)* Altered cytokines release*. Trained memory responses are characterised by an enhanced protective inflammatory reaction. The different patterns of cytokine release may be involved in the systemic establishment of a memory phenotype, reaching far/secluded anatomical sites (as suggested for brain responses and demonstrated in plants).

The main mechanisms of trained memory are summarised in Figure 1. Whether all these mechanisms are concomitantly involved or which one is mainly responsible for shifting innate immune cells toward a memory-like phenotype is still a matter of investigation.

## 4. Improving Adjuvanted Vaccine Formulations by Exploiting the Concept of Innate Immune Memory

Nonspecific side effects of vaccines are a highly debated topic, as an increasing number of parents refuse to immunise their children, fearing side effects and unforeseeable long-term problems [56, 57]. This worrisome trend compromises herd immunity and can lead to serious disease outbreaks, which would not occur in the case of vaccination compliance. In Europe alone, more than 30,000 measles cases have been registered in 2013 [58]. As already pointed out, the nonspecific effects of vaccination are a fact. In most cases such effects increase and broaden protection, while only in some instances have they caused problems. The nonspecific effects of vaccination should be thoroughly investigated, in order to avoid the adverse consequences and optimise the beneficial effects of vaccines.

Thus, the development of future vaccines should take into account not only pathogen-specific immunity but also the nonspecific effects mediated by innate memory. Several issues should be considered on the contribution of innate immune memory to vaccine formulations:

1. Adjuvants that are already in use and act* via* PRR signalling (e.g., TLRa) possibly hold the potential of inducing innate memory and could thereby mediate long-term changes in host defense. Particular attention should be paid to potential variability of reaction depending on sex, ethnicity, and age.
2. Boosting innate defense mechanisms through trained memory induction seems particularly appealing for vulnerable populations that show impaired resistance to pathogens in general. However, boosted nonspecific immunity might also have beneficial outcomes on herd immunity in an average population against widespread diseases, such as the common cold.
3. PAMPs that are able to robustly induce trained memory might also feature potential adjuvant capacity.
4. Enhancing nonspecific effects induced by vaccination can affect the immune response to other routine immunisations, modulating the antibody titre and improving overall protective response, as seen for BCG vaccination [22].
5. Sequence/timing and combination of vaccines against different pathogens are very important aspects of vaccination programmes. Importantly, detrimental nonspecific effects have been noted only when an inactivated vaccine was the most recent one [31]. Thus, changing the current vaccine policies with an improved schedule of vaccinations could be advantageous to avoid negative side effects of vaccines and fully exploit their potential benefits [32, 59].
6. Induction of trained immune memory might improve the induction of specific protection by low-efficiency vaccines.
7. Nonspecific effects of established vaccines have to be further investigated in order to determine their potential in long-term innate immune memory.
8. The memory-inducing capacity of a vaccine might depend on various factors (e.g., the microorganism growth rate) during the vaccine production process.
9. Well-known vaccines with beneficial nonspecific effects could be (re)introduced in countries where they are not part of the immunisation schedule.

## 5. Concluding Remarks

The increased awareness of the properties of innate memory is changing our understanding of host defense and immunological memory and could lead to defining new classes of vaccines and adjuvants. Two major aspects have to be fully addressed, the in-depth identification of the molecular and cellular mechanisms involved and the duration of protection provided by innate memory, which is lifelong in plants and insects but not well evaluated in mammalian systems. Both epigenetic and metabolic reprogramming can be induced during establishment of innate memory. No information is however available on the possible cross-talk and cross-regulation between these events.

Several questions are still open, concerning the epigenetic memory upon infection or vaccination. Does an epigenetic inheritance during myeloid cell linage division exist? Can epigenetic reprogramming be maintained during cell differentiation or upon reinfection? How long lasting are the memory reprogramming effects? Future studies will shed light on these open questions.

A better understanding of innate memory mechanisms in general, and of those induced by licensed and candidate adjuvants and vaccines in particular, will help us to exploit in full the beneficial potential of vaccination and reduce all possible side effects.

## Figures and Tables

**Table 1:** Innate memory versus adaptive memory.

Innate memory		Adaptive memory
Organisms	Plants, invertebrates, vertebrates	Higher vertebrates

Cell types	NK cells, monocytes, macrophages	B and T lymphocytes

Mechanisms	Functional re-programming (e.g., epigenetic modification)	Antigen-specific antibodies and receptors after gene rearrangement

Duration	Medium- to long-term (?)	Long-term

Specificity	No (?)	Yes

Protection	Broad	Limited, highly specific

**Table 2:** Definitions.

*Adaptive Memory* Adaptive memory is long-term, antigen-specific ability of T and B lymphocytes to respond more rapidly and more efficiently to a specific antigen upon second encounter.	

*Innate Memory* Innate memory is the ability of an organism to adapt its immune response depending on a previous infections or vaccination, mediated by NK cells and monocytes/macrophages. This immunological re-programming can result in non-specific suppression (tolerance) or increased innate immune response (training) against reinfection by the same or different pathogens.	

*Trained Immunity/Memory* Trained immunity/memory is the enhanced nonspecific protection against infections after previous exposure to certain microbial components (e.g., *β*-glucans), possibly involving epigenetic and metabolic re-programming in the cell.	

*Tolerance* Tolerance is the refractory state of monocytes/macrophages, involving epigenetic remodelling, induced by microbial components (e.g., LPS). Upon subsequent challenge, even with a high dose of LPS, a less robust induction of pro-inflammatory cytokines ensues.	

*Nonspecific Effects* “Nonspecific” immune effects are induced by a vaccination or infection, against unrelated and antigenically diverse infectious agents. Nonspecific effects are mediated by cross-reactive lymphocytes and innate memory cells, and might be either beneficial or detrimental, depending on the type of memory of the cells involved.	
